# Taking the Lag out of Jet Lag through Model-Based Schedule Design

**DOI:** 10.1371/journal.pcbi.1000418

**Published:** 2009-06-19

**Authors:** Dennis A. Dean, Daniel B. Forger, Elizabeth B. Klerman

**Affiliations:** 1 Division of Sleep Medicine, Brigham & Women's Hospital, Boston, Massachusetts, United States of America; 2 Department of Mathematics and the Center for Computational Medicine and Biology, University of Michigan, Ann Arbor, Michigan, United States of America; 3 Harvard Medical School, Boston, Massachusetts, United States of America; University College London, United Kingdom

## Abstract

Travel across multiple time zones results in desynchronization of environmental time cues and the sleep–wake schedule from their normal phase relationships with the endogenous circadian system. Circadian misalignment can result in poor neurobehavioral performance, decreased sleep efficiency, and inappropriately timed physiological signals including gastrointestinal activity and hormone release. Frequent and repeated transmeridian travel is associated with long-term cognitive deficits, and rodents experimentally exposed to repeated schedule shifts have increased death rates. One approach to reduce the short-term circadian, sleep–wake, and performance problems is to use mathematical models of the circadian pacemaker to design countermeasures that rapidly shift the circadian pacemaker to align with the new schedule. In this paper, the use of mathematical models to design sleep–wake and countermeasure schedules for improved performance is demonstrated. We present an approach to designing interventions that combines an algorithm for optimal placement of countermeasures with a novel mode of schedule representation. With these methods, rapid circadian resynchrony and the resulting improvement in neurobehavioral performance can be quickly achieved even after moderate to large shifts in the sleep–wake schedule. The key schedule design inputs are endogenous circadian period length, desired sleep–wake schedule, length of intervention, background light level, and countermeasure strength. The new schedule representation facilitates schedule design, simulation studies, and experiment design and significantly decreases the amount of time to design an appropriate intervention. The method presented in this paper has direct implications for designing jet lag, shift-work, and non-24-hour schedules, including scheduling for extreme environments, such as in space, undersea, or in polar regions.

## Author Summary

Traveling across several times zones can cause an individual to experience “jet lag,” which includes trouble sleeping at night and trouble remaining awake during the day. A major cause of these effects is the desynchronization between the body's internal circadian clock and local environmental cues. A well-known intervention to resynchronize an individual's clock with the environment is appropriately timed light exposure. Used as an intervention, properly timed light stimuli can reset an individual's internal circadian clock to align with local time, resulting in more efficient sleep, a decrease in fatigue, and an increase in cognitive performance. The contrary is also true: poorly timed light exposure can prolong the resynchronization process. In this paper, we present a computational method for automatically determining the proper placement of these interventional light stimuli. We used this method to simulate shifting sleep–wake schedules (as seen in jet lag situations) and design interventions. Essential to our approach is the use of mathematical models that simulate the body's internal circadian clock and its effect on human performance. Our results include quicker design of multiple schedule alternatives and predictions of substantial performance improvements relative to no intervention. Therefore, our methods allow us to use these models not only to assess schedules but also to interactively design schedules that will result in improved performance.

## Introduction

Endogenous circadian (∼24 hour) rhythms are important physiological regulators of sleep quality and duration, hormone levels, mood (including alertness), and cognitive neurobehavioral performance in humans [1]. The significant effect of circadian timing (phase) on performance has been shown experimentally (e.g., [2]–[6] and in epidemiologic studies of accidents [7]–[17]. Changes in light exposure, sleep-wake patterns, and circadian rhythms associated with jet lag, space travel, and some work schedules have profound effects on multiple physiologic systems, including performance [1], [18]–[23]. The phase and amplitude of endogenous circadian rhythms, generated by a self-sustained pacemaker in the hypothalamus, are affected by ocular light stimuli [24],[25]. Therefore light stimuli have been used to shift the circadian pacemaker to be aligned with a new sleep-wake schedule, resulting in an increase in subjective alertness and objective performance at desired times compared with schedules without properly timed light pulses [2],[26].

Ocular light stimuli can accelerate the re-entrainment of the circadian system with the new sleep-wake schedule [27]–[33] or maintain circadian entrainment [34]–[37]. Many characteristics of light are important: the wavelength, timing, intensity, and duration of a light pulse all have non-linear effects on the magnitude and direction of a circadian phase shift [31], [38]–[43]. Even the intensity of indoor light can have significant impact on the circadian phase of individuals [44]. In addition, because of non-linear photic processing by the retina, intermittent light exposure is disproportionately effective relative to a continuous light exposure: light stimuli that comprise 23% of the time during a total stimulus length of 6.5 hours resulted in phase resetting 74% of that observed after light exposure during the entire 6.5 hours [31].

This non-linear circadian rhythm response to light stimuli [41], [45]–[47] means that it is difficult to develop general rules for designing interventions or countermeasures (CMs) that facilitate re-entrainment to a shifted sleep-wake cycle. Therefore, a mathematical model of the effect of light on the circadian pacemaker is required to accurately predict the non-linear relationship between light input and the resulting circadian phase and amplitude. Mathematical models of the circadian pacemaker and its effects on performance and alertness have been used for at least 20 years. The models aim to predict performance for a range of experimental and operational schedules or applications [48],[49]. To distribute and use these models, specialized software has been developed [48],[49], resulting in a wider range of individuals accessing and using these mathematical models. However, previous work has used the models only to evaluate the effect of light and sleep-wake schedules on circadian phase or performance. There has been very little work done on developing systematic methods for designing schedules or CMs. Herein, we advance the functionality of a mathematical model of the effect of light on the circadian pacemaker and a model of circadian effects on performance to design CMs that facilitate re-entrainment of the circadian pacemaker and therefore optimal performance following a shift in sleep-wake schedule. In this paper, we present a framework for using mathematical models of the human circadian pacemaker and performance to automatically design ocular light stimuli as CMs for a user-defined schedule in which the sleep-wake or work schedule is not at optimal circadian times. While this example uses light as the CM, the methods that we have derived can be used for other CMs, such as pharmaceuticals [1],[50], and for other physiological systems affected by the circadian system (e.g., endocrine concentrations instead of performance). The method includes the development of a new mode of schedule representation that allows for schedule optimization problems to be quickly specified and solved within an analytical and computational framework.

Designing a schedule with optimal CMs presents multiple challenges. (1) Specifying CM location, duration, and intensity can be a combinatorially difficult problem: as the number of days to optimize increases, the number of possible CM placements increases exponentially, making the computation of all possible schedules intractable for long schedules if the method used involves systematic search of possible solutions. (2) Each schedule may have additional scheduling constraints (e.g., specific work tasks must occur at predetermined times; light CM must occur during the waking day; sleep episodes must be 8 hours in duration). (3) Each schedule is evaluated with a non-linear mathematical model. With a non-linear model, small changes in the input (schedule *design*) can result in varying changes in output (*prediction* of circadian phase and performance).

One possible approach to framing the CM design problem is to seek a single solution based on minimizing a specific metric. Optimization of light input to the circadian pacemaker has been approached through the calculus of variation [51] and model-based predictive control [52],[53]. Both approaches provide a technique for determining an analytical solution to the optimization problem. Most notably, one group has demonstrated the use of control theory techniques to evaluate multiple molecular controls to a circadian clock in a non-linear control framework [54]. Our approach and subsequent problem definition differs from a purely optimization approach and emphasizes schedule design. Rather than seeking a single solution, the methods presented aim to develop a framework for allowing schedule/experiment designers to explicitly explore tradeoffs between design parameters such as light duration and intensity, because they may be flexible in the operational setting. Hence, our method allows for multiple solutions to be determined while providing mechanisms for maintaining scheduling constraints.

The time required to manually manipulate and simulate schedule variations limits the number of schedules that can be evaluated. The time spent on schedule *design* can be attributed to: (1) entering complicated and long sleep-wake schedules into the models, and (2) satisfying a dynamic set of scheduling constraints, such as scheduling specific events relative to each other. Consider a 24.65 hour “day” as experienced by ground-based employees working on Mars-related missions, such as the 2008 NASA Phoenix mission. These 24.65-hr “days” are outside the range of circadian entrainment for many individuals under low light intensity levels (<40 lux) and without a light CM [36],[55],[56]. An obvious question to ask of the mathematical models is what light level would be required to maintain entrainment or to ensure high levels of performance at operationally significant times (e.g., during launch or landing). One way to answer this question is to change the light levels at different times within each wake episode and rerun the protocol until a result is achieved. This exhaustive search simulation process (usually involving manually manipulating schedule parameters) has been used successfully to design experimental protocols or operational schedules, and has resulted in insights into the response of the circadian pacemaker to different stimuli [31],[35],[57]. However, manual analysis of schedules that include multiple possible changes in scheduled sleep-wake and multiple possible changes in timing and intensity of light as done in a study of humans living on a non-24-hour day [57] may require several weeks. Therefore, manual manipulation of schedules is not conducive to CM or schedule optimization projects.

We define the light CM *design* problem as follows: given an operational schedule, determine the timing, intensity, and duration of a CM so that circadian phase is aligned with the new sleep-wake schedule to optimize sleep, alertness, and performance, as required. To solve this design problem, we present a new algorithmic method - the circadian adjustment method (CAM) - that can be used to quickly and effectively design light CM for jet lag or shift-work or other shifted sleep schedules and for extreme environments (e.g., space, aquatic, earth poles) that include low light levels and non-24-hour cycles. To allow for families of designs to be generated, CM strength (duration and intensity) are set according to user design constraints (i.e., available hardware light intensity, available time for light exposure). The CAM then determines optimal placement given the user-specified CM strength. Thus, our method allows for both user-specified parameters (e.g., intensity and duration) and algorithmically determined parameters (e.g., timing).

To illustrate our algorithm, we design an intervention for a 12-hour shift in sleep-wake schedule; this phase shift is similar to what an individual would experience in traveling from, e.g., New York to Hong Kong. This shifting problem was selected because it is both theoretically (the maximum that can occur on earth) and operationally significant. In the operational setting, both absolute performance and the duration for which performance levels can be maintained are important. Therefore, the measures of interest were speed of circadian phase adjustment, quartiles of absolute performance within each waking day and across days, relative changes in performance quartiles, and the cumulative probability distribution of performance.

## Methods

### Mathematical model

**Figure 1:**
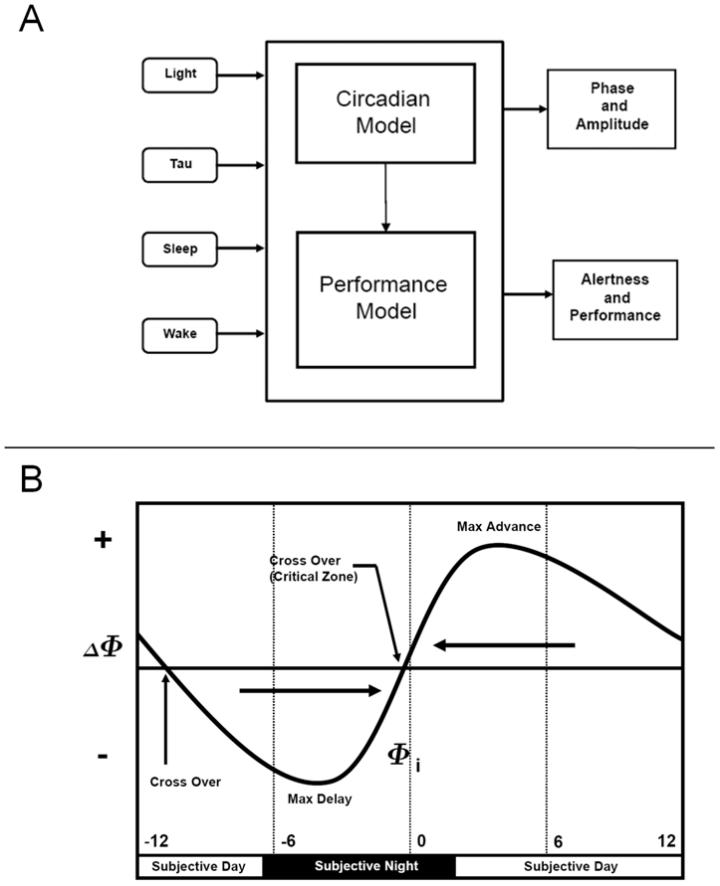
Schema of the mathematical model and the simulated PRC to light. *Panel A*. A schematic of the circadian and performance/alertness mathematical models [58],[59]. Both light intensity and endogenous period (“tau”) are inputs to the circadian model to make predictions of the phase and amplitude of the circadian pacemaker. The inputs to the neurobehavioral models are the sleep-wake times and the output of the circadian model. The outputs of the performance models include subjective alertness and objective performance measures. *Panel B*. Schematic of a phase response curve (PRC) to light stimuli. Circadian phase in hours (Φi) is displayed on the x-axis. Circadian Phase = 0 corresponds to the time of the minimum of the core body temperature, an accepted circadian phase marker. The y-axis displays the change in circadian phase (ΔΦ) ( =  phase after stimulus minus phase before stimulus (Φi)) following a light countermeasure centered at Φi. The PRC consists of two regions: a phase delay (negative phase shift) and a phase advance (positive phase shift) region. If a light stimulus occurs in the delay region, the subsequent circadian phase will occur at a later clock time; the opposite is true for the advance region.

We used a mathematical model of the effect of light on the circadian pacemaker and a linked mathematical model of the effects of the circadian system and sleep-wake state on neurobehavioral performance and alertness [58],[59]. Each component of these models reflects physiological processes. A schematic of the models is shown in Figure 1A. The model of the effect of light on the circadian pacemaker uses modified Van der Pol oscillator equations, with endogenous circadian period and light intensity as a function of time as input. The model then predicts circadian phase and amplitude [58],[59]. The model's phase and amplitude predictions have been experimentally correlated with established circadian markers (e.g., [31],[40],[41],[60],[61]). Figure 1B illustrates the general relationship between the timing of a light pulse and the direction and magnitude of the subsequent phase shift, producing a “phase response curve” (PRC).

In the linked mathematical model of neurobehavioral performance and alertness, the key components are circadian, homeostatic, and sleep inertia functions. The circadian component is the component of performance that is modulated by circadian phase and amplitude; its values are determined from the model of the effects of light on the human circadian pacemaker. The homeostatic component models the effect of time asleep or awake on performance. More specifically, the homeostatic component of performance specifies the decrease in performance during wake and the recovery of performance during sleep. Lastly, the sleep inertia component models the transient low levels of alertness or performance observed immediately after awakening. Sleep inertia is the grogginess experienced immediately after awakening. Performance and alertness values are scaled between 0 and 1, with 1 being the maximum possible performance. The overall structure of the performance and alertness models are the same, although the equations are different for each performance or alertness measure [58]. This work has been validated with data collected in extended wake and non-24-hour experimental protocols [58],[62],[63]. For brevity, only the “performance” model for a serial addition task will be used in this manuscript. The mathematical models can be summarized in a functional form as follows:
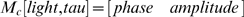
(1)

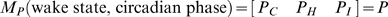
(2)

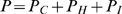
(3)where 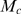 represents the circadian model, 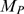 represents the performance model, 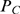 represents the circadian component of performance, 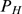 represents the homeostatic component of performance, 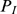 represents sleep inertia, and 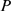 represents overall performance. Although, the components are described separately in the equations above, there is a non-linear interaction between the circadian and homeostatic components in the current formulation of 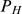 [58]. Note that the functional form of the circadian and performance models is presented to facilitate the specification of our algorithm. Our algorithm assumes that the functional form of the models relates to a set of differential equations that have been validated with experimental data.

### Schedule representation

**Figure 2:**
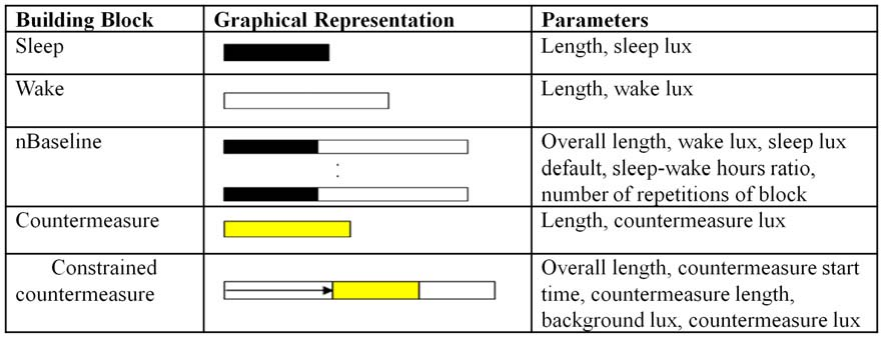
Examples of ‘Schedule Building Blocks’. Note that the constraint in the “Constrained Countermeasure” is assumed to be a timing-related constraint and is therefore instantiated in the countermeasure start time and countermeasure length parameters.

A protocol is defined as a list of events (*e*) that occur sequentially in time. Each event is defined by setting a duration (*d*), light intensity (*l*), and sleep-wake state (σ) as shown in Equations 4–6:
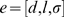
(4)where the sleep-wake state (σ) is defined to be sleep (*s*) or wake (*w*) such that:
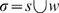
(5)Consequently, a protocol can be defined as a collection of events or as the time-varying vector of duration, light intensity, or sleep-wake state (Equation 6):
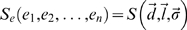
(6)The parameterized form of an event is a schedule building block, which is the schedule primitive used in our representation (Figure 2). It is specified formally as:
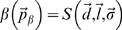
(7)where 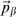 is a vector of parameters:
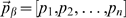
(8)We define a schedule 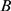 as a list of schedule building blocks:
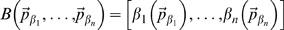
(9)By instantiating (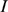) the parameters of a schedule (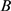), the schedule can be represented as a collection of time-varying vectors (Equation 10):
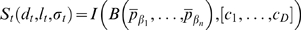
(10)The value of *D* is the total number of parameters for the entire schedule, and *c_i_* represents the current parameter value. By convention, we assume the parameters and the constant values are evaluated from left to right. The schedule representation has been restricted to a regular grammar [64], which is a simple language specification that allows us to specify a simple parser (based on finite state machines) to evaluate the schedule and to convert the schedule into a form suitable for simulation and optimization studies. This schedule building block design allows information regarding clock time and biological time of day (circadian phase predictions) to be used in an optimization framework while maintaining schedule constraints.

### Simulating a schedule

We first simulate the circadian phase and amplitude predictions of that schedule using the mathematical model. We then use these phase predictions to select optimal regions for placing CMs, using the circadian and performance model presented above. To generalize the application of this class of models, we introduce the following notation for predicting circadian phase given a mathematical model (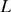), a schedule (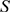), and the endogenous period (τ) of the pacemaker (Equation 11):
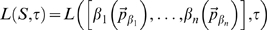
(11)
*L* is the model of the circadian effect of light on the pacemaker, and the schedule is represented as a list of building blocks (eq. 9). Each building block 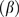 has a variable list of parameters 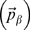, as noted above. We use the time of the core body temperature (CBT) minimum 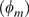, the circadian marker to which this model has traditionally been referenced, for the circadian phase marker. The phase of the CBT minimum can be represented as:
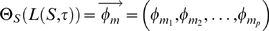
(12)where 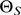 is a function that extracts the model-predicted circadian phase minima per 24 hours from the specified schedule 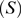 and 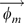 is a vector of discrete CBT minima. The performance model can be compactly represented as a function of the schedule 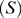 and the prediction of circadian phase 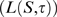.
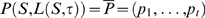
(13)


### Defining the optimization problem

We first compare the baseline phase angle difference (i.e., between predicted 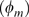 and habitual wake 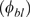) with the predicted phase angle 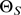 during the shifted sleep episode 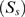. The shifted sleep episode 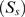 is determined by selecting the sleep events 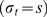. The target phase angle 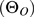 is computed by adding the start of the sleep event 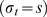 to the length of the sleep event 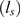 and subtracting the baseline phase angle 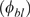:
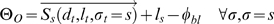
(14)The objective function for this optimization problem is designed to minimize the absolute value of the difference between the predicted phase angle 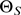 and the target phase angle 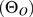.
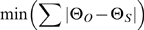
(15)The simulated phase, due to the schedule building block formulation, is a function of the parameters of schedule (S) and the endogenous period of the pacemaker:
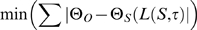
(16)To obtain a closed form of the objective function, Equation 10 is substituted into Equation 16 to yield:
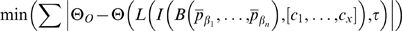
(17)Equation 17 is a compact form of the schedule optimization problem and is a function of schedule parameters and the endogenous period of the circadian pacemaker.

### Circadian Adjustment Method (CAM)

The CAM is an iterative technique that uses information about predicted circadian phase to determine placement of CMs such that the final result is robust and optimal. The steps involved in this technique are:

1. Use Equation 12 to simulate the schedule 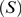 without a countermeasure to obtain an initial phase estimate and compute the predicted circadian phase 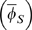.
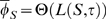
(18)
2. Compute the initial placement of the CM 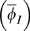 as follows. Set CM placement (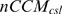) such that the end of the CM precedes the predicted circadian phase by a predetermined constant 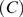 to insure the CM is in the appropriate region of the PRC and simulate the schedule with CMs 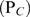. Selecting the constant C to insure convergence is considered in the **Results** section.
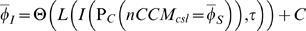
(19)
3. Adjust CM placements 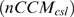 such that they precede the predicted circadian phase marker (from step 2) by C, the predetermined amount. Adjustment insures that the CM placement avoids the Type 0 resetting portion of the PRC [65]. Type 0 resetting is described below.
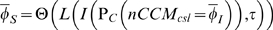
(20)
4. Substitute 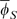 into 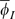 in step 3 and repeat until the phase prediction 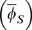 converges or for a fixed number of iterations chosen by the user. (See results section)

The nature of the CAM is to exploit the physiological effect of placing a bright light pulse prior to the CBT minimum, which results in shifting the subsequent CBT minimum to a later clock time. Traditionally, determining the effect of a light pulse is accomplished with a PRC (e.g., Figure 1B) in which the relative phase shift is shown on the ordinate and the timing relative to the CBT minimum (or other phase angle measure) is displayed on the abscissa. Rather than look up values on a static PRC, a mathematical model of the effect of light on the circadian pacemaker is used here. Through simulating the schedule, the mathematical models can be used to generate the phase response based on the lighting conditions.

The mathematical model of the effects of light on the human circadian pacemaker is capable of simulating the experimentally observed Type 0 response to light, which includes traversing a singularity region in phase space, similar to being exactly at the north or south pole, which has no defined longitude (analogous to no phase/time). The exclusion of the Type 0 resetting solution is part of the overall CAM strategy of obtaining a solution for a single solution space. The exclusion of the Type 0 solution space is further justified because it is technically difficult to achieve Type 0 resetting, and a phase shift in the wrong direction could easily occur from a slightly mistimed stimulus in this region. Further details of the convergence characteristics of the CAM are presented in a computational proof of convergence in the Supporting Information (Text S1).

### Software

*Shifter* is a prototype scheduling software constructed to use the schedule building blocks in conjunction with the CAM to design and optimize schedules. Its implementation includes the formalism and nomenclature presented above for models, schedules, simulation mechanics, and the CAM.

*Shifter* was implemented in MATLAB version 7.7 (Natick, MA). *Shifter*'s graphical user interface was developed with Guide (MATLAB's graphical user interface development tool). The schedule building blocks are implemented as MATLAB functions. Each building block is designed to be called with a variable number of parameters. The optimization interface is designed to use both the CAM and Nelder-Mead (MATLAB's fminsearch function) to allow schedules with a variable number of parameters to be optimized. Additional details of *Shifter*'s functionality are presented in the Supporting Information (Text S1, Figure S3, Figure S4, and Figure S5).

## Results

### Verification of stability of phase delay region

**Figure 3:**
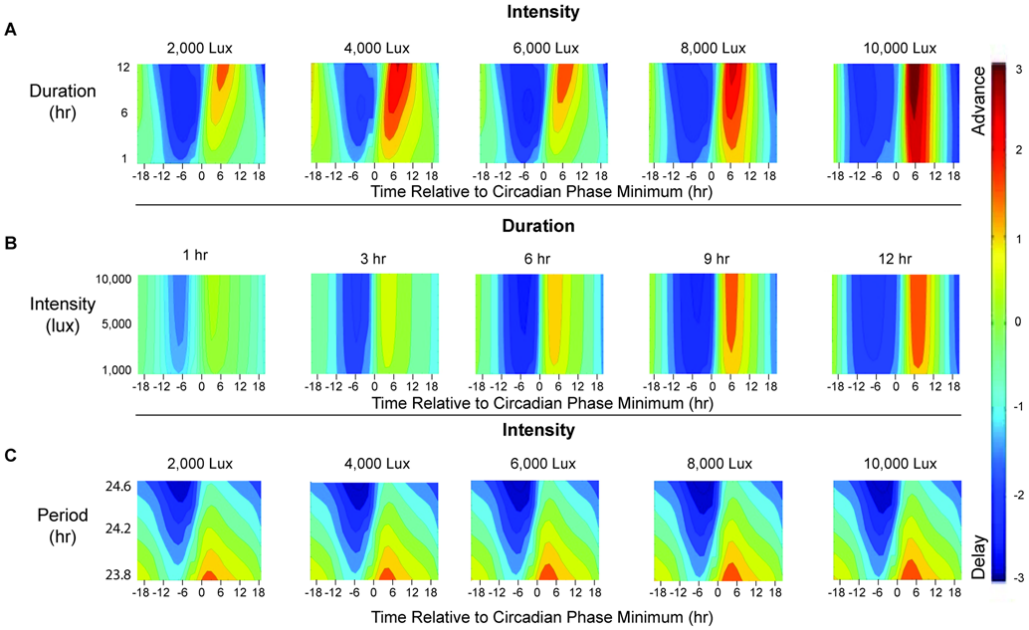
Phase response contours from simulations of phase response protocols. The horizontal axis represents the timing of the countermeasure center (in hours), relative to the time of the predicted core body temperature minimum (Circadian Phase = 0). The vertical axis represents the specific parameter being studied: duration (Panel A), intensity (Panel B), endogenous circadian period (Panel C). The magnitude of the phase shift (in hours) is color coded according to the legend. The maximum delay and advance regions are colored dark blue and dark red, respectively. *Panel A*. Duration (1 to 12 hr) response contours for light pulses with different intensities (1,000–10,000 lux). Simulations were run with an endogenous period of 24.2 hr. *Panel B*. Intensity (1000–10,000 lux) response contours for different light pulse durations (1–12 hr). Simulations were run with an endogenous period of 24.2 hr. *Panel C*. Endogenous period (23.8–24.6 hr) response contours for different intensities (1,000–10,000 lux). Simulations were run with 3-hr light pulse durations.

The Circadian Adjustment Method (CAM) requires stable phase advance and delay regions. This condition was verified with phase response contour maps that were created from 3240 simulations using the mathematical model of the effects of light on the human circadian pacemaker (see **Methods**) of phase response protocols with two-way combinations of varying CM duration (1–12 hr), CM intensity (1,000–10,000 lux), and endogenous circadian period (23.8–24.6 hr) (Figure 3). The phase response protocol is a standard chronobiology technique for assessing the response of the circadian system to a scheduled light stimulus [41]. The phase response protocol contains three sections: 1) The pre-stimulus section contains an 8-hour sleep episode followed by a wake episode. The length of the wake episode ranges from 28 hours to 52 hours so that the scheduled CM (see section 2) can be placed at any phase of the circadian system. 2) The stimulus section contains an 8-hour sleep episode followed by a 16-hour wake episode. 3) The post-stimulus section contains an 8-hour sleep episode followed by a variable length wake episode. The length of the post-stimulus wake episode is selected to insure that the duration of the entire phase response protocol is constant. The shift in circadian phase (reported in Figure 3) is calculated as the difference in predicted circadian phase in the post- and pre-stimulus sections. As shown in Figure 3, the phase *regions* of maximum delay and advance are relatively constant. The relative stability of the phase delay region supports the use of a constant offset (parameter C in Equation 9; See **Methods**). The plots also demonstrate that the circadian system has a larger amplitude for phase delay responses than for phase advance responses to light stimuli of different durations and intensities.

### Defining schedule representation and the CAM

Designing optimal CMs requires a flexible and extendable method for specifying schedules that includes an analytic link. The CAM uses “building blocks” (Figure 2) to represent arbitrary schedule components and relationships between these components. These schedule building blocks have two key features: (1) They include a set of parameters that can either be fixed by the user or defined as a variable for subsequent analysis, including optimization. Note that the constraint in the “Constrained Countermeasure” is assumed to be a timing-related constraint and is therefore instantiated in the countermeasure start time and countermeasure length parameters. (2) They are constructed in a way that allows parameter values to change during the optimization process. Thus the schedule building block formulation allows for explicit (e.g., light CM presented during the waking day) and implicit constraints (e.g., 8-hour scheduled sleep episode) to be maintained. For this problem, we use five different types of schedule building blocks (Figure 2). Although each building block contains many parameters, only CM intensity, duration, and placement were considered for this problem. The mathematical details of this schedule representation are described in **Methods**.

We tested optimal control theory, gradient descent methods, and direct search methods [53],[66]. Gradient descent and direct search methods do not provide robust solutions, due to the presence of multiple solutions (phase delay, phase-shifting through the singularity, phase advance) and the nonlinearities of the mathematical models. We also sought solutions to the boundary value problem that resulted by applying the calculus of variations to the mathematical models [51]. The boundary value problem did not converge to a solution in operationally significant conditions. Consequently, we sought a solution that had robust convergence characteristics (targeting a single solution) and did not require simulating every possible combination of schedule parameters. We therefore developed the CAM (details in **Methods**).

To test the ability of the CAM to converge to a unique result without user intervention, the method was applied to a range of CM durations (1, 3, 5, and 7 hours) and intensities (500, 750, 1000, 2500, 5000, and 10,000 lux) for the test protocol (one 24-hour baseline day followed by a 12-hour phase shift, which includes a 28-hour wake episode, followed by 12 24-hour days). Each of the 24 solutions (4 durations×6 intensities) converged within 5–20 iterations.

To evaluate the performance of the CAM, a Nelder-Mead optimization procedure [67],[68] was initialized with the results from the CAM and applied to this test problem. The Nelder-Mead optimization method is a direct search method that has been used in a variety of optimization problems. For this test problem, the value of the objective function without a CM was 220.2 hr which represents the total number of hours the predicted circadian phase is misaligned from the target circadian phase making the value of the objective function an index of circadian misalignment that can be used to compare schedules. Following the optimization of light CM with the CAM, this was reduced to 50.45 hr; and was further reduced to 49.90 hr following the Nelder-Mead optimization procedure (Table 1). Therefore, the CAM, when augmented with the Nelder-Mead procedure, is a robust and near optimal solution to this CM design problem.

**Table 1:** Changes in objective function values following optimization procedures.

	Objective Function	Number of Function Calls	Change in Objective Function	
			Absolute Decrease	% Decrease
Without a countermeasure	220.2	1	-	-
Circadian Adjustment Method (CAM)	50.5	11	169.8	77%
CAM followed by Nelder-Mead	49.9	61	170.30	77%

*: lower is better for value of objective function.

The CAM used in conjunction with Nelder-Mead can be viewed as a two-step optimization procedure. The effectiveness of the CAM is contingent on the proper selection of C in Equation 19. Through simulations (see above) we have determine that a near optimal region can be obtained by specifying a constant offset (C in equation 9) from the predicted minimum of CBT. Using the CAM output as an input to the Nelder-Mead procedure provides a method to finely tune the placement prediction. Thus, we use an optimization scheme tailored for our specific problem to find a near optimal solution and fine tune with a general optimization scheme. The simulation studies in Figure 3 demonstrate that increasing light intensity and duration increases the magnitude but does not change the direction of the phase shift without substantially changing the location of the optimal timing of the CM. Additional examples of using light intensity and duration to design schedules are in the Supporting Information (Text S1, Figure S1, and Figure S2).

### Simulated results

**Figure 4:**
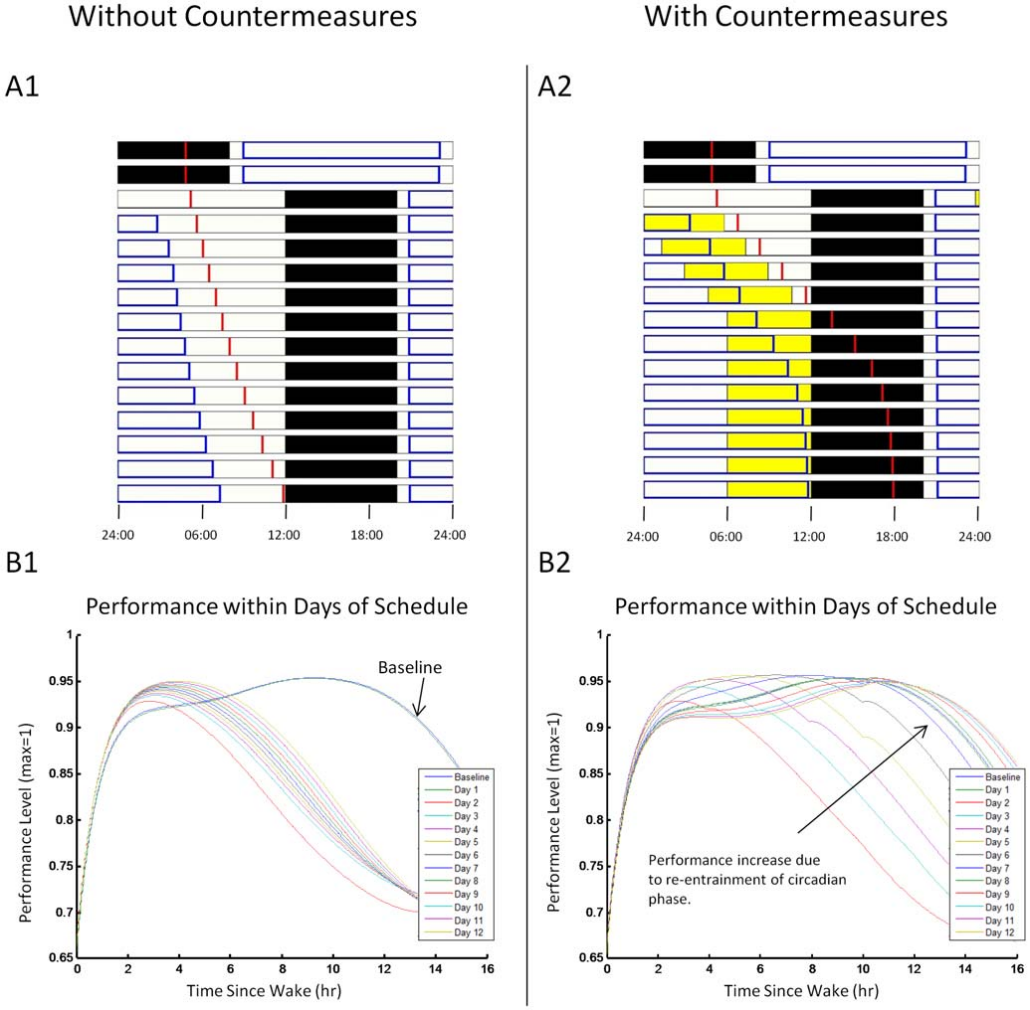
Schedule and simulation results of a jet-lag schedule. The schedule includes two baseline days, a 12-hour shift in scheduled sleep episode, followed by 12 days at the new schedule. Panels A1 and B1 are the simulations without a countermeasure; Panels A2 and B2 are the simulations with a countermeasure. *Panels A1 and A2*. Raster plots of the schedule and simulation results: time (midnight to midnight) is represented horizontally, and each line is a separate day. Black boxes represent the timing of sleep episodes, white boxes represent the timing of wake episodes, yellow rectangles represent the timing of the bright light countermeasure, blue rectangles represent times of >85% performance, and red vertical lines represent time of predicted core body temperature minimum (the marker of circadian phase). The target phase used in the objective function is shown by the light blue vertical line in the shifted sleep. *Panels B1 and B2*. The performance within each wake episode across all days of the schedule is shown without (B1) and with (B2) countermeasures; each color represents a different day of the protocol. As circadian phase moves closer to the target phase, there is a higher level of performance for a longer duration each day. The countermeasure speeds this phase shift and results in faster improvement in performance, especially after ∼6 hours of wake.

During entrained baseline conditions, simulations predict the circadian phase zero (time of CBT minimum) to be 2.8 hours prior to habitual wake time. After the 12-hr phase shift of the sleep-wake schedule in the test protocol (Figure 4A), there are marked changes in circadian and performance measures. Without a CM, circadian phase (in relation to the new schedule) slowly changes, but never achieves the same timing relationship with the sleep episode as during baseline conditions (Figure 4-A1). In contrast, simulation of the schedule with a CM resulted in reestablishment of the entrained circadian phase relationship following 8 days of the CM (Figure 4-A2). Predicted performance during each wake episode has a sharp initial rise (Figure 4-B1 and 4-B2), consistent with the decay of the sleep inertia component. The ability to maintain levels of performance >85% of maximum during the waking day is reduced from 12 hours during baseline to 6.5 hours without a CM. (Figure 4-B1). In contrast, simulation results of the schedule with a CM resulted in faster recovery of performance levels within each day and across successive days (Figure 4-B2). By day 6, the performance profile across the day is similar to that during baseline conditions for CM but not for no-CM conditions. (Figure 4-B2).

Illustrations of the effects of CM strength are included in the Supporting Information (Text S1 and Figure S1).

### Quantifying performance changes

**Figure 5:**
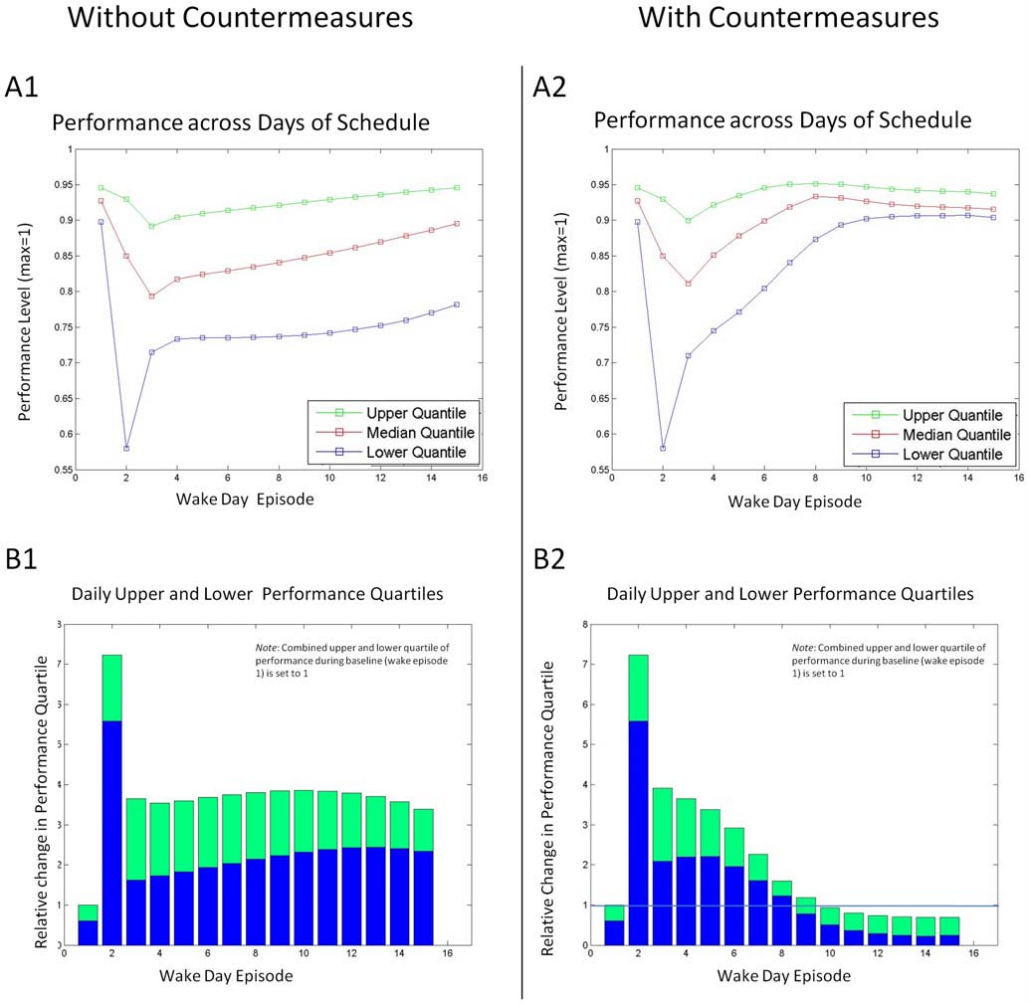
Simulated changes in daily performance with and without countermeasure after a jet-lag schedule. The schedule is the same as in Figure 4. Panels A1 and B1 are the simulations without a countermeasure; Panels A2 and B2 are the simulations with a countermeasure. *Panels A1–A2*: The predicted performance upper quartile (green), median (red), and lower (blue) quartile for each wake episode across all days of the schedule. *Panels B1–B2*: The scaled upper and lower quartiles across wake episodes of the schedules. For panels B1–B2, the combined upper (green) and lower (blue) quartile of simulated performance during baseline (wake day episode 1) is scaled to 1.

Quartiles of the range of performance values, rather than mean and standard deviation, were used to evaluate the schedules for two reasons. First, the performance predictions during the waking day do not have a statistically normal distribution. Second, the lower quartile of performance, during a day, was a more sensitive indicator of entrained circadian phase (Figure 5-A2) and may also be more operationally relevant. Thus, here we are more concerned about improving the lowest levels of performance (which have been attributed to many catastrophic errors [49]) than the mean level of performance.

During baseline, the performance quartile values (25%, 50% (median), and 75%) are 0.90, 0.93 and 0.95, respectively. Note the maximum predicted performance is 0.95 during the baseline day, which is a consequence of the data scaling procedure specified in Jewett [62]. The performance quartile values during the 28-hour extended wake episode (day 2) that accompanies the 12-hour phase shift are lower than the median performance during the baseline day (0.58, 0.85, 0.92), and the 0.34 units difference between the upper and lower quartiles of performance is nearly seven times that of the baseline day (0.05 units), mostly due to the dramatic decrease in the lower quartile value. Following the extended wake episode, there was approximate symmetry of the upper and lower quartiles around the median. Without a CM, performance improves slowly over each waking episode for the next 12 wake episodes after the schedule shift. The time course of recovery for the performance quartile values is approximately linear with the number of recovery days (Figure 5-A1). While the upper quartile and median values reach baseline levels after 12 days, the lower quartile value remains at 86% of baseline and 75% of maximum performance. Between days 3 and 15, the difference between the combined upper and lower quartile of performance over the waking day remains constant at four times the combined quartile range during the baseline day (Figure 5-A1 and 5-B1).

With a CM, the performance quartile values reach >90% of baseline values after 8 days and return to baseline levels after 9 days (Figure 5-B2). In addition, the difference between daily upper and lower quartile ranges decreases linearly for each of the first 6 days following the application of the CM (Figure 5-B1) and then decreases rapidly to an asymptote.

### Empirical cumulative probability function

**Figure 6:**
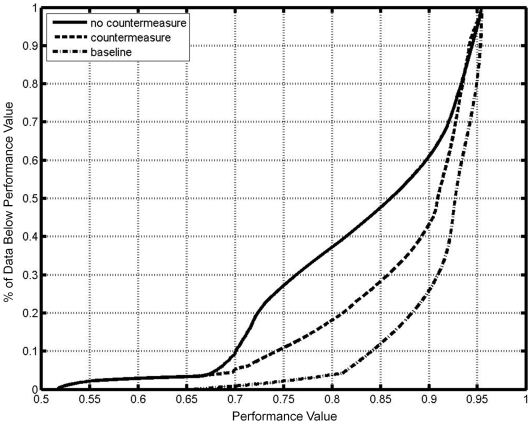
The empirical cumulative probability distribution of performance. The distribution is shown for baseline (dot-dashed), and across the entire protocol with (dashed) and without (solid) a countermeasure.

We also compared the empirical cumulative probability function of performance during the baseline day and for the entire protocol for CM and no-CM conditions (Figure 6). This cumulative probability function demonstrates the percentage of time that simulated performance is below a chosen value. The baseline condition had a higher percentage of time at higher simulated performance levels than the CM and no-CM conditions. The percentage of the waking day above a simulated performance level of 0.80 for the baseline, CM and no-CM conditions were 95%, 80% and 60%, respectively.

## Discussion

The primary contribution of this work is an efficient and practical approach to designing re-entrainment schedules that uses both a novel schedule representation (schedule building blocks) and a novel algorithm for locating optimal solutions (circadian adjustment method, CAM). Our algorithm provides advantages over existing circadian schedule design techniques that evaluate a large number of schedules (genetic algorithms, enumeration) or use existing optimization techniques (Nelder-Mead, gradient descent, optimal control theory). Enumeration of all possible schedules quickly becomes computationally intractable as the number of days in the schedule increases. Existing optimization techniques are generally formulated to extract one solution that may be unrealistic in the operational setting. Our algorithm has been designed specifically to allow for multiple solutions to be determined through the specification of design and optimization parameters. The schedule design parameters (i.e. light level, light duration, sleep length) allow for families of schedules to be considered, which is analogous to facilitating constrained enumeration through the use of schedule building blocks. Consequently, a key contribution of the method is the integration of the schedule representation with an optimization approach, which gives the advantage of evaluating a large number of schedules with optimization, while reducing the drawbacks when each approach is used alone.

The CAM is designed to both exploit features of the solution space and to have good convergence characteristics. In practice, optimizing Equation 17 directly is challenging due to multiple solutions to the entrainment problem. The mathematical formulation of the circadian models allows for phase advances, phase delays, and phase jumps through the singularity region (Type 0 resetting) [65]. Phase jumps through the singularity region have been shown experimentally and mathematically. However, the practical difficulty in targeting the singularity regions (to date there is only one experimental demonstration in humans [65]) may make the approach operationally impractical. From an operational design perspective, the ability to choose a particular solution has advantages, since the schedule may contain other phase advance or delay characteristics. Consequently, a key feature of the CAM is to select the specific solution region (phase delay or phase advance) in which to optimize. The CAM insures that CM starting values are near the maximum shift of the region, which in turn insures that computational efforts are not wasted in poor solution spaces.

We have shown that a mathematical model of the effect of light on circadian phase and the effects of circadian rhythms and length of time awake on performance can be used to automatically design light CMs to facilitate re-entrainment after a shift in schedule. Our work illustrates that the CM design process can be divided into schedule specification and schedule optimization components. The schedule specification component allows users to define a parameterized schedule and arbitrary schedule constraints. The schedule optimization component optimizes the objective function constructed from user-specified parameters. The method extracts operationally relevant information such as the timing of wake episodes and predicted circadian phase levels from mathematical simulation that is used to optimize CMs.

The scheduled building block formulation of the CAM is an iterative procedure whose functional form is motivated by the lambda calculus [69]. A practical benefit of the lambda calculus specification is a precise and unambiguous implementation prescription, through functional programming methods [69], that outlines the transition of the algorithm to software. It also allows formal analysis (convergence, running time, memory requirements). Moreover, the nomenclature and formalism provide standard interfaces for which to study different schedules, different optimization methods, and different models. The formalism and hence the software implementation are designed to evolve as new models, methods, and schedules are considered. Thus, a major goal of the formalism is to provide a mechanism to maximize the use of existing software implementation and minimize the amount of software development required for studying different aspects of schedule design.

The iterative procedure converges quickly for a variety of operationally relevant conditions. The method results in a substantial reduction of design time compared with manual analysis, which, in our experience, reduces the design of intervention schedules from the order of days to minutes. The convergence and computationally efficient characteristics of our methods are suitable for interactive design of schedules. Recall that our test problem was to determine the duration, intensity, and placement of light that facilitates re-entrainment of the circadian system. Our system has both user-specified (duration and intensity) and algorithmic (placement) parameters. The user pre-sets the CM duration and intensity based on operational constraints. The CAM then determines the CM placement for optimizing re-entrainment. Since the algorithm generally convergences in less than two minutes on a laptop computer, the methods can be used to interactively design, evaluate, and compare several alternative designs (e.g., different durations and intensities) in real time [70]–[72].

Although we used a simple test example, our methodology could easily be expanded to include different shifting strategies. For example, one strategy in the literature is to use light as a CM to advance the schedule prior to phase delaying [73]. To search for the appropriate advancing schedule, we would have to change the instructions in step 3 of the CAM to place the light pulse just after the CBT minimum, as determined by the PRC to light. Our method, therefore, is easily adjustable so that studies of schedules with both advances and delays could be determined and evaluated.

Whereas in this work light was used as the CM, the methodological framework was designed to be easily extended to include different CMs, such as naps, caffeine, or other pharmaceutical agents. The only requirement is that a phase response curve for that CM exists. A planned addition to the work is computing confidence intervals for the CM placements and performing a sensitivity analysis on schedule parameters. A general statistical framework for comparing alternative schedule designs, determining schedule parameter confidence intervals, and computing parameter sensitivity will also be important.

### Implications of results on schedule design

These simulations have multiple implications for schedule design. (1) Schedules that use CM at the time of greatest effect result in faster re-entrainment of the circadian system. Under entrained conditions, CBT minimum (the time of maximum sensitivity to light stimuli, see Figures 1B and 3) occurs during sleep, approximately 2 hours before scheduled wake. Therefore, light exposure as a CM at this circadian-sensitive time can only occur when sleep timing is shifted. (2) While the magnitude of phase advances are nearly equal to that of phase delays (Figure 3-A), the narrow maximum phase advance region may be an impractical target for operational environments. (3) The difference between upper and lower quartiles of performance (Figure 5-B) may be a strong indicator of circadian entrainment. Examining quartiles of performance may be an appropriate surrogate for circadian entrainment which is currently not possible to assess in real time in the operational setting. Analysis of experimental and field data is required to validate this prediction. This method may also be valuable in determining the number of days a CM is required. For example in Figure 5, note that, with a CM, on day 11 the difference between upper and lower quartiles returns to the baseline value. In subsequent days, the difference falls to below that of baseline. A plausible interpretation of this finding is that CMs are only required up to day 11. Applying CMs on future days may not only be unnecessary and costly in time and responses but may also result in further, undesired, changes in the relationship between predicted circadian phase and the wake episode (Figure 4-A2). An example of the effect of inappropriately timed bright light pulses is in the Supporting Information (Text S1 and Figure S1).

Estimating the light levels is an important aspect of using these models. We have found that reasonable modeling predictions can be made with limited information about background light for indoor and outdoor conditions (Dean, personal communication). The ability to use averaged light levels is a direct consequence of the underlying mathematical models and is due to the non-linear response of the circadian pacemaker to light. Consequently, only the order of magnitude of the light-level is important for these simulations [59]. The light preprocessor in our model also acts as a low pass filter, smoothing (in the time domain) the light information input to the pacemaker.

In practice, the intrinsic circadian period parameter can be used to design group and individual interventions, since intrinsic period length is normally distributed [74]. When the individual circadian period has been determined experimentally, the measured or derived (e.g., from other physiologic measures such as the phase relationship between circadian phase and sleep-wake schedule [75]) intrinsic period should be used and will result in an individualized design of light placement.

Several aspects of the schedule design problem warrant further study: (1) formal methods for embedding schedule constraints, (2) alternative objective functions, (3) initializing and optimizing schedule parameters, and (4) statistical methods for comparing and evaluating schedules. In future work, the current building block formulation of the CAM will be expanded to incorporate additional scheduling components and constraints, allowing for a range of schedule optimization problems to be studied.

### Implications of results for other computational problems

The novelty of this work is the coupling of schedule representation that facilitates both maintaining constraints and optimization in a modular format. The representation of the problem within a “building block” (Figure 2) that can be optimized is the core of the work. We anticipate that these methods can be generalized for use with other optimization problems that have inherent constraints (operational and biological) and with other optimization methods. Our aim in developing a specific module for jet lag is to demonstrate the efficacy of our framework and the computational advance. Our future work will proceed in two directions. The first is to develop modules (schedule building blocks and corresponding mathematical models) for optimizing additional CMs including melatonin [76],[77]. Properly timed melatonin is effective in shifting the circadian system. The second area of work will be to enhance the schedule building block formulation to include additional operational-related constraints and countermeasure design strategies.

Our simulation studies show that, when timed correctly, CM light intensity and duration affect the magnitude of the shift in circadian phase (Figure 3). Consequently, the CAM emphasizes the optimization of pulse placement without regard to pulse duration or intensity. Bright light strength (duration and intensity) can then be used as design variables to adjust for differences in available lighting hardware, conflicts of scheduled bright light exposure time with other operational activities, and personal preferences in acceptable bright light strength.

## Supporting Information

Text S1 Supplemental Material
(0.06 MB PDF)

Figure S1 Simulations demonstrating the effect of intervention placement and strength in facilitating adaptation of the body's internal circadian clock to a shift in sleep/wake timing.
(0.37 MB TIF)

Figure S2 Simulations of non-24-hour-day schedules.
(0.35 MB TIF)

Figure S3 Shifter screen shot showing a schedule with and without designed countermeasure.
(1.66 MB TIF)

Figure S4 Examples of user-defined schedules and interventions generated with Shifter.
(0.68 MB TIF)

Figure S5 Predicted performance summaries generated with Shifter.
(1.63 MB TIF)
