# Supplementary material for: Taking the Lag out of Jet Lag through Model-Based Schedule Design
Source: PLoS Comput Biol. 2009 Jun 19;5(6):e1000418. doi: 10.1371/journal.pcbi.1000418 (PMC2691990; doi:10.1371/journal.pcbi.1000418)
Supplement: Text S1 — Supplemental Material (0.06 MB PDF) [file pcbi.1000418.s001.pdf]

Supplemental Material:

A. Proof of Convergence

In this Appendix, we provide a computational proof that the circadian adjustment method (CAM) belongs to the class of fixed-point iteration schemes (FPIS) [1] and therefore will converge to a valid solution. One Definition and Two Theorems are required for this proof. *Definition 1* and *Theorem 1* are from Bradie [1], which also contains the proof of Theorem 1. Thomas et. al contains the proof of Theorem 2.

Definition 1: A fixed point iteration scheme to approximate the fixed point, p, of a function g, generates the sequence $\{p_n\}$ by the rule $p_n = g(p_{n-1})$ for all $n \geq 1$ given a starting approximation, p_0.

Theorem 1: Let g be continuous on the closed interval $[a,b]$ with $g:[a,b] \to [a,b]$. Furthermore, suppose that g is differentiable on the open interval (a,b) and there exists a positive constant $k < 1$ such that $|g'(x)| \leq k < 1$ for all $x \in (a,b)$. Then

1. *The sequence* $\{p_n\}$ *generated by* $p_n = g(p_{n-1})$ *converges to the fixed point* p *for any* p_0 *element in* $[a,b]$
2. $|p_n - p_{n-1}| \leq k^n \max(p_0 - a, b - p_0)$ *and,*
3. $|p_n - p| \leq \dfrac{k^n}{1-k}|p_1 - p_0|$

Theorem 2: A composition of continuous functions is continuous [2].

Proof of convergence of CAM methods:

Step 1: Show that the CAM satisfies the definition of a fixed-point iteration scheme (FPIS). (Def. 1). Equation 20 from the manuscript can be converted to the form $p_n = g(p_{n-1})$ by substituting $\bar{\phi}_n$ and $\bar{\phi}_{n-1}$ respectively for $\bar{\phi}_S$ and $\bar{\phi}_I$ resulting in the expression (S1). Note that the intrinsic period τ is a constant.

$$\bar{\phi}_n = \Theta\Big(L\Big(I\left(\mathrm{P}_C\left(nCCM_{csl} = \bar{\phi}_{n-1}\right)\right), \tau\Big)\Big) = g\left(\bar{\phi}_{n-1}\right) \tag{S1}$$

Step 2: Show that the CAM satisfies the criteria in Theorem 1

For convergence of equation (S1) to hold, (1) g must be continuous, (2) g must be differentiable, and (3) the magnitude of the derivative of g is less than or equal to k, where k is less than 1 in the interval [a,b] as required in Theorem 1.

Definition*:* Let the interval $[a,b]$ be defined during the wake episode such that *a* corresponds to the waking time and *b* corresponds to circadian phase (CBT min). The variable *x* corresponds to a time between *a* and *b*

Definition/Assumption*:* A single maximum phase delay exists within [*a*, *b*].

We will restrict the proof to a circadian phase delay (positive shift). A similar proof/construction would be made for a phase advance without loss of generality. For simplicity, only a single countermeasure placement is considered. The requirements for convergence will be considered separately.

(1) Continuous. The function *g* in equation S1 represents a composition of 4 functions $(\Theta, L, I, \mathrm{P}_C)$. By Theorem 2, for *g* to be continuous, each of the functions must be continuous in the interval considered. Two of the functions (I, P_C) of the CAM (details in main manuscript) are translation functions that convert the input into a form suitable for simulation, and one function (Θ) converts the simulation output into a form suitable for computing the next iteration of countermeasure placements. These three translations will be continuous if the fourth function (*L*) is continuous, since the other three functions translate the schedule and the continuous output into a form suitable for simulation. Since the schedule is fixed except for a countermeasure that can be placed along the continuous variable x, the input is continuous.

We define *L* as the phase response space depicted in Figures 1 and 3 of the main manuscript. The phase response space is generated from a limit cycle model that contains a singularity and hence multiple solutions. However, since by definition, we are only using the phase delay or phase advance region, the limit cycle equations are continuous in the defined interval. The contour plots in Figure 3 of the main text demonstrate numerically that the simulated contour maps are continuous. In addition, experimental and analytical evidence suggests that using light to shift circadian phase through the singularity is an extremely unlikely event [3,4].

(2) Differentiable. Since only the continuous region of the phase response is considered, the derivative of the phase response exists for the specified region.

(3) The value of the magnitude of the derivative g is less than or equal to k, where k is less than 1 in the interval [*a*,*b*]. We will first demonstrate that the equivalent expression is one in which subsequent changes in predicted circadian phase approach zero as the number of iterations increases. It can then be shown that there exists the required k with a value less than 1.

Definition: The final desired phase time *f* is defined as the *x* corresponding to the maximum predicted shift of the circadian phase given a light stimulus.

The CAM is initialized to $\bar{\phi}_I$ which is set arbitrarily to a distance prior to the predicted circadian phase minimum (because we are restricting this work to phase delays) and is also set prior to the distance *c*, which corresponds to the maximum phase shift possible.

Assumption. It is assumed that there exists a single maximum delay for a given set of light intensities and durations along x. This assumption is supported through simulation and experimental evidence (see main manuscript).

The restrictions imposed by this assumption are that the background and countermeasure light levels are sufficient for entrainment. In addition, the day length is assumed to be entrainable. (See Supplemental Materials for additional detail). This restriction insures that a solution exists (i.e., schedule is entrainable).

We will show by contradiction that the distance between the final predicted circadian phase minimum and the prior predicted phase approaches zero as the number of iterations increases.

Lemma. Let *d* = the distance between predicted circadian phase and the final (desired) circadian phase (*f*). Then all subsequent d must be less than or equal to the initial distance between predicted circadian phase and the final (fixed) circadian phase d_0.

This lemma can be shown true by contradiction. If there exists a *d* greater then d_0 at least one of the following must be true: 1) a phase advance must have occurred, 2) the position of *a* must have shifted to the right, or the position of *f* must have shifted to the left. However, if each were true, then the result would 1) violate construction of the CAM (advances are not allowed), 2) contradict the definition of *a* being fixed by construction, or 3) contradict the definition of *f*.

Lemma. The sequence $\{d_n\}$ approaches 0 as the number of iterations (n) increases.

This lemma can be shown true by demonstrating that subsequent applications of light push the predicted core body temperature minimum to the right, which results in *d* decreasing. At d_0 the light pulse is applied at a position in x that is less then optimal by construction. Due to the phase response of light in the delay

region, the position of d_1 along x will be closer to *f*. This argument holds true for all subsequent iterations of *d*.

Lemma. If the distance between subsequent *d* is zero, then the fixed point has been achieved. True by construction.

The remainder of the proof follows from the proof in Bradie [1]. Since at each iteration *d* approaches zero, there exists a k that is less than 1. After *n* iterations the term *k* is repeated resulting in the expression k^n which approaches zero as *n* goes to infinity. Hence, we have shown that the value of the magnitude of the derivative g is less than or equal to k, where k is less than 1 in the interval $[a,b]$

We have shown that the CAM meets the FPIS requirements for convergence (Theorem 1). Consequently, the CAM is a fixed-point iteration scheme and it converges.

References

1. Brian Bradie (2006) Fixed Point Iteration Scheme. In: A Friendly Introduction to Numerical Analysis. Upper Saddle River: Pearson Education, Inc. pp. 81-94.

2. George B.Thomas Jr, Ross L.Finney (1988) Calculus and Analytical Geometry. Addison-Wesley Publishing. 1136 p.

3. Indic P, Forger DB, St.Hilaire MA, Dean DA, Brown EN, Kronauer RE, Klerman EB, Jewett ME (2005) Comparison of amplitude recovery dynamics of two limit cycle oscillator models of the human circadian pacemaker. Chronobiol Int 22: 613-629.

4. Jewett ME, Kronauer RE, Czeisler CA (1991) Light-induced suppression of endogenous circadian amplitude in humans. Nature 350: 59-62.

B. Examples of Designing Light Interventions with the Circadian Adjustment Method (CAM)

Example 1: Understanding the effect of light intensity and duration on countermeasure placement

Appropriately placed ocular bright light exposure can be used to assist the adaptation of the human internal circadian timing system to a shift in sleep-wake timing. Additional examples of different light exposure patterns using the sample sleep/wake schedule in the main manuscript are presented here to demonstrate how lighting affects adaptation to a shift in sleep/wake schedule.

No Intervention (Figure S1:A1-3): The background light in the No-Intervention case of Figure S1A is set to 5 lux and there is no bright light countermeasure scheduled. This background light level is relatively low but is similar to that of some work environments, such as that of a rail operator working at night. At low light levels and without a bright light intervention (countermeasure), the circadian system does not receive enough timing cues to facilitate adaptation to the new sleep-wake schedule. The lack of adaptation is shown by the presence of the predicted core body temperature minimum during the waking day (Figure S1-A1), instead of during the scheduled sleep episode (which is the optimal timing, since performance and alertness are worst at the time of the predicted core body temperature minimum). The consequence is that the predicted daily performance levels return to baseline slowly (Figure S1-A2) and the inter-quartile (25% - 75%) difference is between 3 and 4 times the inter-quartile difference at baseline (Figure S1-A3) throughout.

Poor Intervention (Figure S1: B1-3): A bright light exposure can be inappropriately timed for a schedule. Light pulses (3 hr duration and 1000 lux) are placed during the waking day without regard to the predicted circadian phase. For the first part of the schedule, the predicted core body temperature minima shift towards the shifted sleep episode as desired to improve performance. However, due to the initial poor placement of the bright light, near the end of the schedule the bright light stimuli are timed during and after the predicted core body temperature minimum, resulting in the shifting of the temperature minimum away from the scheduled sleep episode. The result of the intervention is not effective in resetting the predicted circadian phase minimum to during the sleep episode. Neither daily performance nor the scaled performance quartiles change appreciably during the intervention. The predicted daily performance levels (Figure S1-B2) and interquartile differences (Figure S1-B3) demonstrate that the scheduled bright light pulses are inadequate to facilitate adaptation to the shifted sleep episode. The poor intervention example is the primary motivation for the development of the CAM.

Appropriate Intervention (Figure S1: C1-3 – E1-3): The next three examples are presented to demonstrate that appropriate bright light countermeasures shorten the number of days required for the circadian clock to readjust to the new sleep-wake schedule. For each example the CAM determined the optimal countermeasure placement and hence the largest expected improvement of performance (Figs. S1-C2, D2, E2) and the largest decrease in interquartile differences (Figs. S1-C3, D3, E3) of expected performance levels given the strength of the intervention.

Short Duration – High Intensity (Figure S1: C1-3) The two-hour 10,000-lux bright light pulses are effective in shifting the predicted core body temperature minimum to occur during the sleep episode, resulting in an increase in daily performance levels and a decrease in scaled interquartile differences.

Long Duration – Low Intensity (Figure S1: D1-3) The five-hour 3000-lux pulses are effective in shifting the predicted core-body temperature minimum to occur during the sleep episode, which results in an increase daily performance and a decrease in interquartile differences.

Long Duration – High Intensity (Figure S1: E1-3) The five-hour 10,000-lux pulses are highly effective in shifting the predicted core body temperature minimum to occur during the sleep episode, resulting in a recovery of daily performance and a decrease in interquartile performance levels.

Example 2: Designing non-24-hour schedules

Non-24-hour schedules are found in some extreme environments (submarine or maritime, space, shift work) and are substantial challenges to the human circadian system. Two schedule variations are presented to demonstrate conditions where designing light countermeasure interventions can and cannot facilitate adaptation to the non-24-hour period. In the first variation the day is lengthened to 24.62 hours, which corresponds approximately to the length of the Mars day. Although this schedule presents what is seemingly only a modest daily change in the sleep-wake schedule, adapting to the schedule is not possible without a light intervention (Figure S2-A1) for most individuals [5,5]. Neither the daily performance quartiles (Figure S2-A2) nor the normalized quartile variations reach a constant level during the schedule; they vary as circadian phase moves into and out of appropriate alignment with the sleep-wake schedule. With a light countermeasure, (Figure S2: B1-B3), entrainment is possible and performance improves.

For the 28-hour day, simulations demonstrate it is not entrainable even with a light intervention. Without a light intervention the predicted circadian phase oscillates at the intrinsic circadian period (24.2 hours) (Figure S2: C1), and performance quartiles and normalized quartiles oscillate as the predicted circadian phase enters and leaves the scheduled sleep episode (Figure S2: C2-C3). Scheduled light intervention (maximum strength) results in only minor differences, demonstrating that the schedule is beyond the limits of human entrainment (Figure S2: D2-D3).

C. Shifter - A tool for Evaluating and Designing Schedules

Shifter Overview

Shifter is a graphical user interface for the mathematical equations described in the manuscript. Its purpose is to demonstrate how schedules and countermeasures can be evaluated and designed to improve predicted performance. *Shifter's* graphical user interface contains parameters and features for designing and optimizing schedules. There are four sections to the A section of the interface: 1) protocol

specification, 2) circadian properties, 3) optimization parameters, and 4) summary and analytical operations. A screen shot of the interface with output is in Figure S3.

1) Protocol Specification. The protocol specification section allows the user to set protocol design parameters such as the amount of background light, waking day length, and sleep episode length. These specific protocol parameters were selected to demonstrate that, with a modest number of parameters, a wide range of schedules can be generated as shown in Figure S4.

2) Circadian Property. The circadian properties allow the user to set the intrinsic circadian period. The default is the average human circadian period (24.2 hr) [6,6,6].

3) Optimization Parameters. The current version of the software has been designed to optimize the placement of light by the use of the circadian adjustment method (CAM), described in the text. The user chooses the intensity, duration, and number of iterations of the light countermeasure. Countermeasure strength (intensity and duration) are user-set design parameters because they are frequently constrained by user requirements (available hardware or schedule issues). Note that placement ("timing") will be optimized with the CAM.

4) Summary and Analytical Operations. *Shifter* produces several graphical outputs that can be used to assess and compare different schedule designs and optimization results. Simulations and optimization actions automatically generate (1) raster plots (B and C sections, Figure S4) that include sleep-wake state, lighting information, simulated performance values, and simulated circadian phase; and (2) continuous plots with time on the x axis and performance level on the y axis(D and E sections). The user can select additional summaries including daily performance quartiles, daily changes in predicted variance, daily performance box plots, and the distribution of simulated performance. Examples of other plots that can be generated by *Shifter* are shown in Figure S5.

References:

1. Brian Bradie (2006) Fixed Point Iteration Scheme. In: A Friendly Introduction to Numerical Analysis. Upper Saddle River: Pearson Education, Inc. pp. 81-94.

2. George B.Thomas Jr, Ross L.Finney (1988) Calculus and Analytical Geometry. Addison-Wesley Publishing. 1136 p.

3. Indic P, Forger DB, St.Hilaire MA, Dean DA, Brown EN, Kronauer RE, Klerman EB, Jewett ME (2005) Comparison of amplitude recovery dynamics of two limit cycle oscillator models of the human circadian pacemaker. Chronobiol Int 22: 613-629.

4. Jewett ME, Kronauer RE, Czeisler CA (1991) Light-induced suppression of endogenous circadian amplitude in humans. Nature 350: 59-62.

5. Gronfier C, Wright Jr. KP, Kronauer RE, Czeisler CA (2007) Entrainment of the human circadian pacemaker to longer-than-24h days. Proc Natl Acad Sci USA 104: 9081-9086.

6. Czeisler CA, Duffy JF, Shanahan TL, Brown EN, Mitchell JF, Rimmer DW, Ronda JM, Silva EJ, Allan JS, Emens JS, Dijk DJ, Kronauer RE (1999) Stability, precision, and near-24-hour period of the human circadian pacemaker. Science 284: 2177-2181.

Supplemental Figure Legends

Figure S1: **Simulations demonstrating the effect of intervention placement and strength in facilitating adaptation of the body's internal circadian clock to a shift in sleep/wake timing. (Left Column)** The schedule and simulated results are shown in a raster plot where each horizontal segment represents 24 hours. The timing of the sleep episode (black), scheduled day with moderate light intensity (100 lux)(white), waking day with dim light (5 lux)(gray), bright light intervention (varies in lux)(yellow), >85% simulated performance regions (blue), and predicted circadian core body temperature minima (red) are shown for each schedule. For C1-D1-E1, light placement is determined by the circadian adjustment method (CAM). **(Middle Column)** Simulated daily performance quartiles (25%-blue, 50% - red, 75% - green) for the schedules in the left column. **(Right Column)** Scaled upper quartile (green) and scaled lower quartile (blue) of simulated performance. The combined the upper and lower quartiles during the baseline day (8 hours sleep, 16 hours wake) is scaled to one.

Figure S2: **Simulations of non-24-hour-day schedules.** Results with (B, D) and without (A, C) a bright light countermeasure intervention are shown. **1)** Each raster plot shows the schedule and simulated results. Symbols are as in Figure S1. **2)** Daily performance quartiles are shown for each schedule. Colors as in Figure S1. **3)** The daily normalized difference in quartiles of predicted performance are shown for each schedule. Colors as in Figure S1. **A, B)** A 24.62-hour (Martian) day **C, D)** A 28-hour day

Figure S3: ***Shifter* screen shot showing a schedule with and without designed countermeasure. A)** The graphical user interface allows for different classes of parameters to be set, including: protocol parameters, circadian properties, countermeasure design parameters, and parameters for summarizing results. **B)** Simulation results for a 12-hour shift in sleep-wake schedule. Each line in the raster plot represents 24 hours of predictions. Black bars represent scheduled sleep episodes, blue rectangles represent >85 percent performance levels, red lines represent predicted circadian phase=0. **C)** Simulations for a 12-hour shift in sleep-wake with a designed countermeasure. Color scheme is the same as in (A) with the addition of yellow representing a scheduled pulse of bright light. The design parameters are intensity (10,000 lux) and duration (6 hours) for this example. The placement parameters are then automatically determined with the circadian adjustment method (CAM) described in the text. **D and E)** The simulation results from (B and C) are shown in a continuous format. The x axis represents time in hours since the start of the protocol; the y axis represents the scaled performance (1 = max possible performance). Color scheme is as previously defined, except blue represents the full range of performance.

Figure S4: **Examples of user-defined schedules and interventions generated with *Shifter*.** Simulated results for 24 hours are presented on each line of the raster plot. Black boxes represent a scheduled sleep episode, white boxes represent the waking day, red lines represent predicted core body temperature minimums (circadian phase =0), and blue rectangles represent >85% performance intervals. **A, B)** Prediction of an abrupt change in scheduled sleep-wake episode with (A) and without (B) a scheduled light countermeasure as seen in jet-lag conditions. **C, D)** Predictions of the effect of living on a >24-hour

day with (**C**) and without (**D**) a countermeasure is shown. **E)** Predictions of the effect of living on a schedule that is <24 hours is shown. **F)** An example of a sleep restriction protocol is shown. The schedules differ in two schedule parameters (day length, sleep percentage). Countermeasures (for panels B and D) are placed automatically with the CAM.

Figure S5: **Predicted performance summaries generated with *Shifter*.** Figures can be generated whether the simulation does (**C**, **D**, **F**, and **H**) or does not (**A**, **C**, **E**, and **G**) contain a scheduled countermeasure. **A, C)** Figures display daily predicted performance quartiles (25%, 50%, 75%). **B, D)** Figures display normalized upper (50 to 75%) and lower (25 to 50 %) quartiles. **E, F)** Figures display daily box plots. **G, H)** Figures display the empirical probability distributions of simulated performance values.
