# Supplementary figures and images for: Taking the Lag out of Jet Lag through Model-Based Schedule Design
Source: PLoS Comput Biol. 2009 Jun 19;5(6):e1000418. doi: 10.1371/journal.pcbi.1000418 (PMC2691990; doi:10.1371/journal.pcbi.1000418)

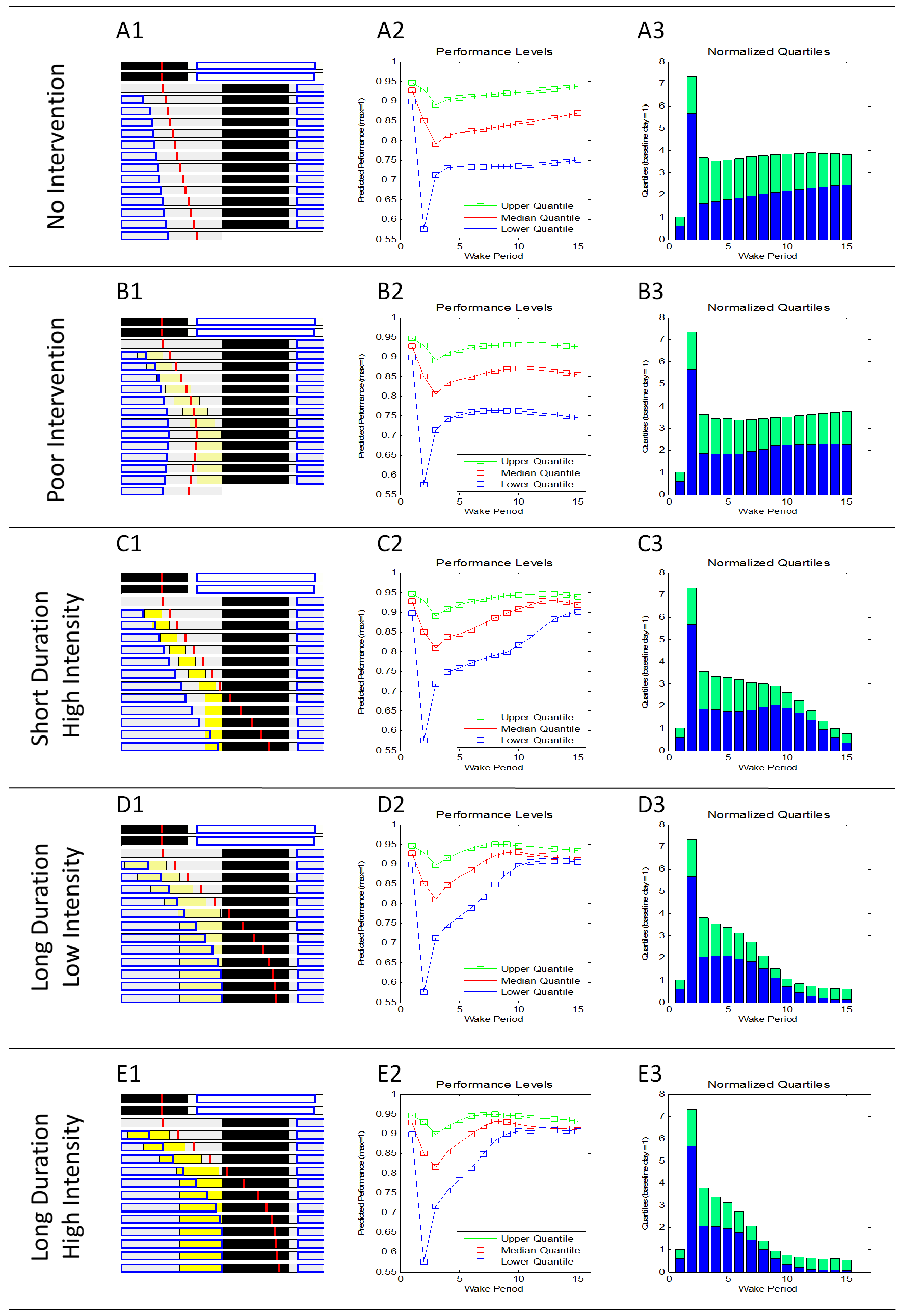

Supplement: Figure S1 — Simulations demonstrating the effect of intervention placement and strength in facilitating adaptation of the body's internal circadian clock to a shift in sleep/wake timing. (0.37 MB TIF) [file pcbi.1000418.s002.tif]

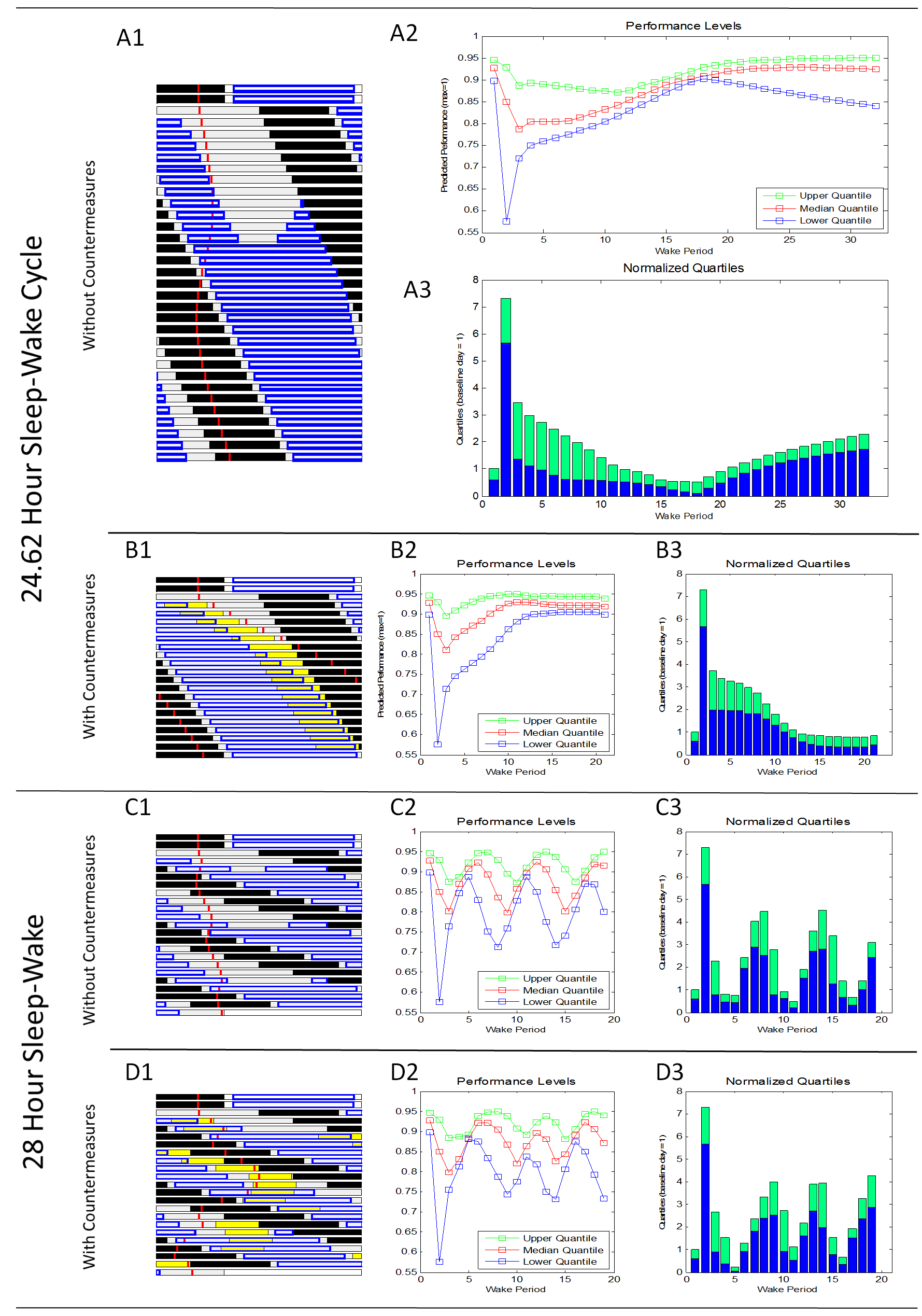

Supplement: Figure S2 — Simulations of non-24-hour-day schedules. (0.35 MB TIF) [file pcbi.1000418.s003.tif]

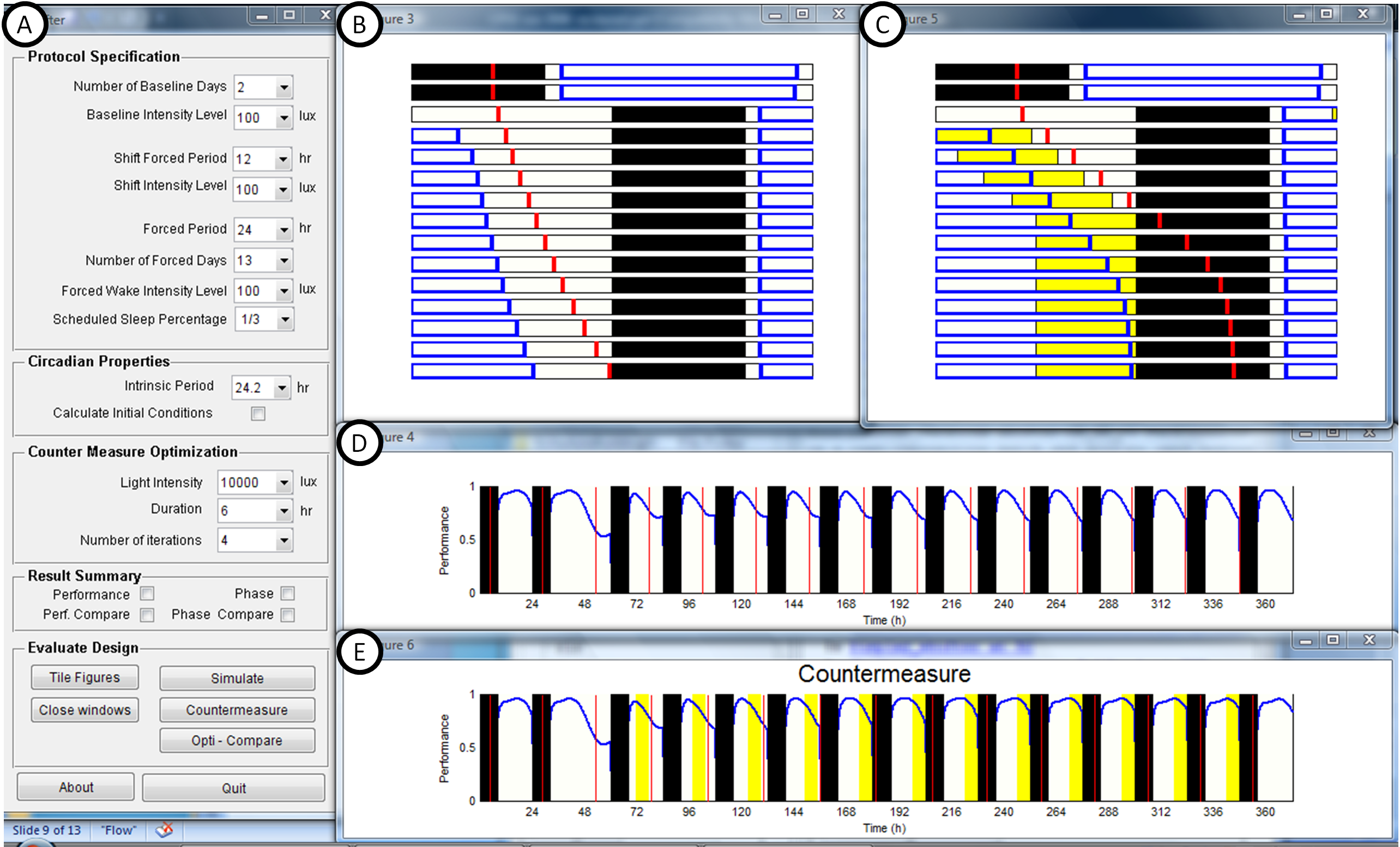

Supplement: Figure S3 — Shifter screen shot showing a schedule with and without designed countermeasure. (1.66 MB TIF) [file pcbi.1000418.s004.tif]

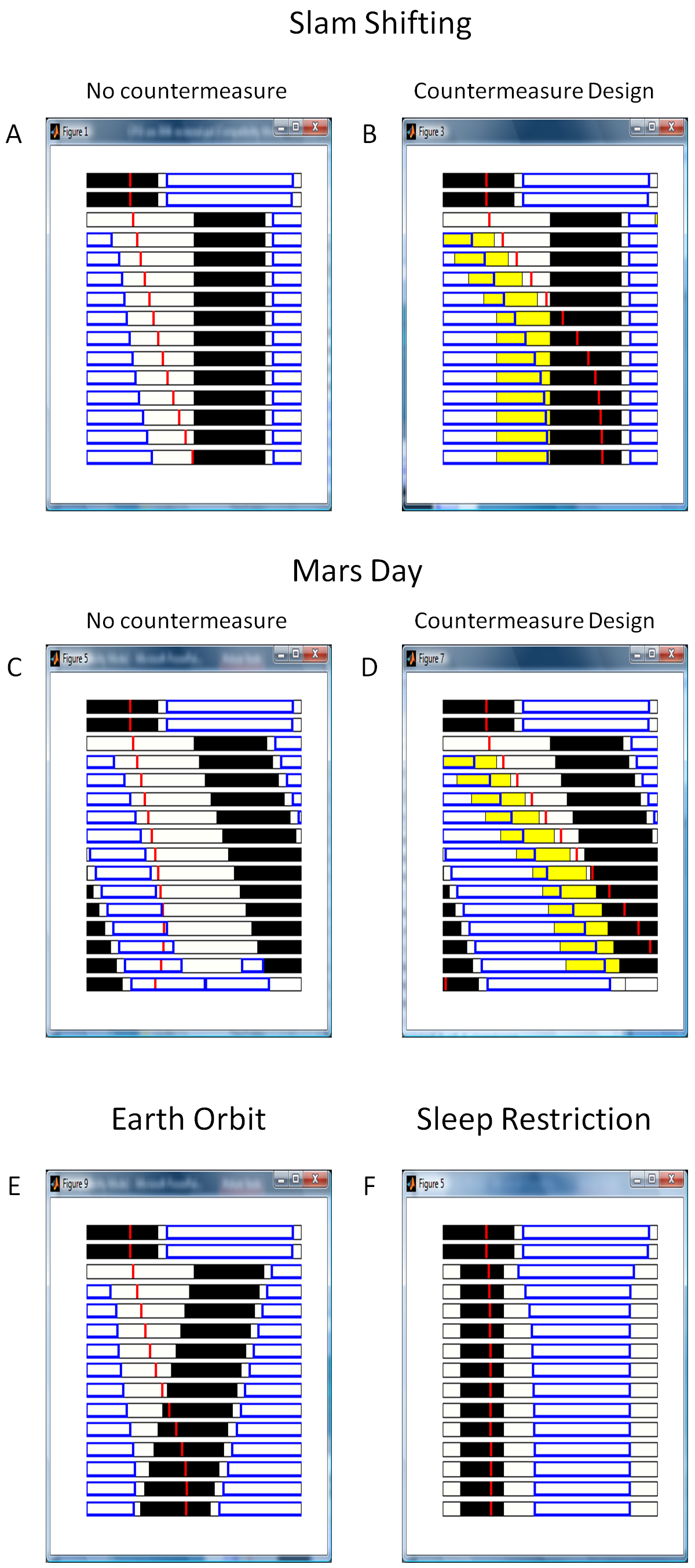

Supplement: Figure S4 — Examples of user-defined schedules and interventions generated with Shifter. (0.68 MB TIF) [file pcbi.1000418.s005.tif]

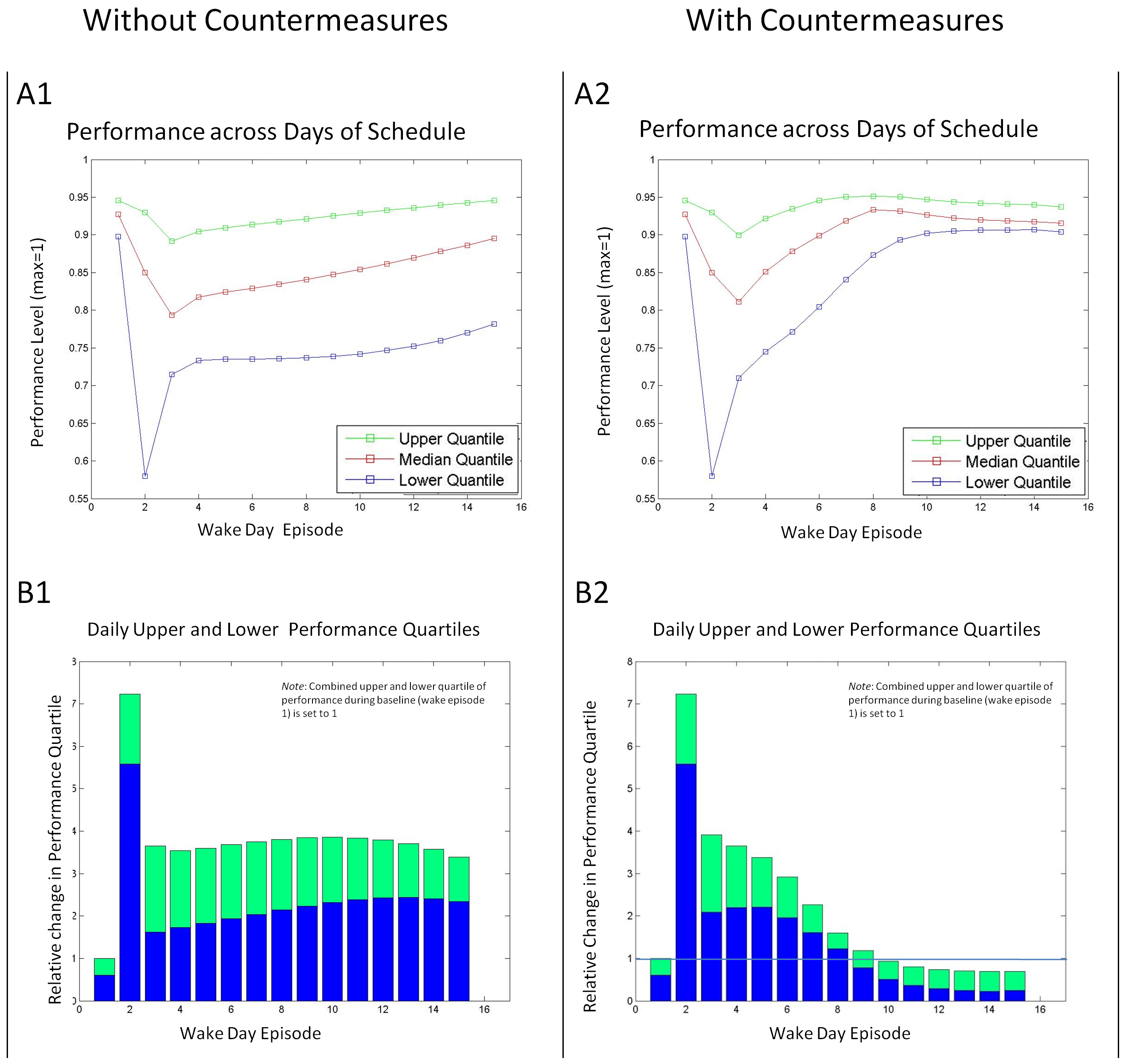

Supplement: Figure S5 — Predicted performance summaries generated with Shifter. (1.63 MB TIF) [file pcbi.1000418.s006.tif]
